# „Homeoffice in Corona-Zeiten – Sind Ausmaß und/oder Flexibilität wichtig für Arbeitszufriedenheit, soziale Unterstützung, Commitment und Arbeitsunterbrechungen?“

**DOI:** 10.1007/s11612-022-00630-z

**Published:** 2022-04-19

**Authors:** Cathrin Becker, Eberhard Thörel, Nina Pauls, Anja S. Göritz

**Affiliations:** Institut für Psychologie, Abteilung Wirtschaftspsychologie, Albert-Ludwigs-Universität Freiburg, Engelbergerstraße 41, 79085 Freiburg, Deutschland

**Keywords:** Homeoffice, Arbeitszufriedenheit, Soziale Unterstützung, Affektives Commitment, Arbeitsunterbrechungen, Teleworking, Job satisfaction, Social support, Affective commitment, Work interruptions

## Abstract

Die Diskussion über Vor- und Nachteile von Homeoffice hat durch die Corona-Pandemie neue Impulse bekommen: Auch Behörden haben ihren Mitarbeitenden die Möglichkeit eingeräumt, ins Homeoffice zu wechseln. Viele Studien zum Thema Homeoffice wurden mit Personen durchgeführt, die schon seit Jahren aus dem Homeoffice arbeiten. Es ist jedoch unklar, welche Konsequenzen ein plötzlicher Wechsel ins Homeoffice haben könnte. In diesem Beitrag wurde in einer Landesbehörde untersucht, inwieweit das Ausmaß an Homeoffice und die Möglichkeit seiner flexiblen Nutzung mit arbeitsbezogenen Variablen wie Zufriedenheit mit der Arbeit, soziale Unterstützung, affektives Commitment und Arbeitsunterbrechungen zusammenhängen. An der Onlinebefragung nahmen 477 Beschäftigte teil. Unsere Analysen zeigen, dass mehr Homeoffice mit weniger Arbeitsunterbrechungen einhergeht. Wir fanden keine signifikanten Zusammenhänge zwischen dem Ausmaß an Homeoffice und Arbeitszufriedenheit, affektivem Commitment oder sozialer Unterstützung. Es gab jedoch positive Zusammenhänge zwischen der Flexibilität des Arbeitsortes mit allen Ergebnisgrößen mit Ausnahme von Arbeitsunterbrechungen. Die Befunde liefern Evidenz für die Bedeutsamkeit der flexiblen Nutzung von Homeoffice und damit der wahrgenommenen Möglichkeit, selbst darüber zu entscheiden, ob im Homeoffice gearbeitet wird. Dies impliziert, dass Organisationen – Behörden inbegriffen – eine mitarbeiterorientierte Flexibilität brauchen, d. h. den Beschäftigten einen möglichst großen Handlungsspielraum bei der individuellen Ausgestaltung flexibler Arbeitsmöglichkeiten geben sollten.

## Abstract (en)

The debate on the advantages and disadvantages of teleworking (i.e., working from home) has gained momentum during the Covid-19 pandemic: Even public authorities have given their employees the opportunity to switch to teleworking. Many studies on teleworking have been carried out with employees who have been teleworking for years. However, it is unclear which consequences a sudden shift to teleworking has. This article examines to what extent the use of teleworking and the possibility of its flexible usage are associated with work-related variables such as job satisfaction, social support, affective commitment and work interruptions. A total of 477 employees of a medium sized public agency took part in the online survey. Our analyses show that more teleworking is associated with fewer work interruptions. We did not find any significant association between the extent of teleworking and job satisfaction, affective commitment and social support. However, there were positive relationships between the perceived flexibility of the place of work with all outcomes with the exception of work interruptions. The findings provide evidence of the importance of the flexible use of teleworking and thus the perceived opportunity to decide for oneself whether to work from home. This implies that organizations—including public agencies—need employee-oriented flexibility, that is, they should give employees the greatest possible decision latitude on the individual use of flexible work options.

## Einleitung

Das Thema Homeoffice ist allgegenwärtig ob bei der Arbeit oder in den Medien. Die Diskussion über Vor- und Nachteile von Homeoffice hat durch die Corona-Pandemie Impulse bekommen (Neumann et al. 2020; Bérastégui 2021). Unternehmen, die bislang gegenüber Homeoffice wenig aufgeschlossen waren, haben ihren Mitarbeitenden erstmals die Möglichkeit gegeben, ins Homeoffice zu wechseln; es gab Debatten über ein Recht auf Homeoffice (z. B. Lott et al. 2021), und manche Beschäftigten machten die Erfahrung, dass die Arbeit von daheim zu ungeahnten Problemen führen kann (Bérastégui 2021). Einerseits haben nun auch Unternehmen und Behörden ihre Mitarbeitenden ins Homeoffice geschickt, die damit bislang zögerlich waren. Andererseits machen manche Beschäftigte jetzt im Homeoffice auch die Erfahrung, dass es sich zuhause oft gar nicht so gut arbeiten lässt wie vielleicht gedacht. Beschäftigte schätzen am Homeoffice zwar, dass sie zu Hause mit weniger Unterbrechungen bestimmte Aufgaben besser erledigen können (Vogl und Kratzer 2015), jedoch verlängert sich die Arbeitszeit eher (Lott 2019) und damit wird die Grenzziehung herausfordernder (Lott et al. 2021). Beschäftigte mit Telearbeitsvereinbarung zeigen ein erhöhtes Risiko der zeitlichen Entgrenzung von Arbeit und Privatleben (Backhaus et al. 2020a).

Doch schon vor der Corona-Pandemie war die zunehmende Bedeutung von Homeoffice, d. h. Arbeit, die von Beschäftigten von zu Hause verrichtet wird (Bundesministerium für Familien, Senioren, Frauen und Jugend 2017), für die Arbeitswelt deutlich geworden: Eine vor der Pandemie durchgeführte Studie zeigte, dass Homeoffice stets eine Arbeitsoption sein sollte, auf die Beschäftigte und Führungskräfte selbstbestimmt und flexibel zurückgreifen können (Kratzer 2020). Spätestens, wenn es in Organisationen, vor allem mit activity based working-Konzepten^1^ an Rückzugsräumen mangelt oder Besprechungszimmer dauerbelegt sind, wird die Möglichkeit zum Homeoffice wichtig (Becker et al. 2021). Damit activity based working-Konzepte funktionieren, brauchen sie Arbeitsplatzoptionen und die Autonomie, diese Optionen auch nutzen zu können. Optionen sind die individuelle Möglichkeit, die räumlichen Bedingungen der Arbeit zu beeinflussen und so die Nutzung des Raumes der eigenen Arbeitsweise und den jeweiligen Anforderungen und Bedürfnissen anpassen zu können. Die Möglichkeit, im Homeoffice zu arbeiten ist hier eine wichtige Arbeitsplatzoption (Kratzer 2020; Becker et al. 2019).

Die Metaanalyse von Gajendran und Harrison (2007) fand Zusammenhänge zwischen Telecommuting – ein Begriff der weiter gefasst ist als Homeoffice und im nächsten Abschnitt erläutert wird – und verschiedenen arbeitsbezogenen Konstrukten. Interessanterweise legten weitergehende Analysen nahe, dass die gefundenen Zusammenhänge teilweise auf die wahrgenommene Autonomie zurückgehen könnten (Gajendran und Harrison 2007). Dies weist darauf hin, dass die Möglichkeit, selbst flexibel zu entscheiden wo man arbeitet, wichtiger sein könnte als der tatsächliche Umfang der Arbeit im Homeoffice. In unserer Studie untersuchten wir daher, inwieweit die Möglichkeit zur Flexibilität und der tatsächliche Umfang der Arbeit im Homeoffice bei einer Landesbehörde mit verschiedenen arbeitsbezogenen Konstrukten zusammenhängen, wenn für die jeweils andere Variable kontrolliert wird. Die Ergebnisse unserer Studie sollen dazu beitragen, die konkrete Ausgestaltung von Homeoffice durch empirische Befunde zu leiten. Beispielsweise würde eine hohe Bedeutung von Flexibilität nahelegen, dass starre Regelungen bezüglich Homeoffice kontraproduktiv wären.

## Theoretischer Hintergrund

Im Zusammenhang mit Homeoffice gibt es verschiedene Begriffe, die teils bedeutungsgleich sind, teils nicht (Ahlers et al. 2021). Im Folgenden werden fünf für diese Arbeit wichtigen Begriffe definiert und im weiteren Text entsprechend verwendet: 1. Telearbeit, 2. mobiles Arbeiten, 3. Homeoffice, 4. Telecommuting, und 5. orts- und zeitflexibles Arbeiten.

Telearbeit ist in der Arbeitsstättenverordnung (ArbStättV) geregelt, mit einem fest eingerichteten Bildschirmarbeitsplatz im Privatbereich der Beschäftigten. Es wird häufig unterschieden zwischen Teleheimarbeit, bei der der Bildschirmarbeitsplatz ausschließlich im Privatbereich der Beschäftigten ist, und alternierender Telearbeit, bei der zwischen dem fest installierten Bildschirmarbeitsplatz in der Betriebstätte und der privaten Wohnung gewechselt wird (Backhaus et al. 2021b).

Der Begriff „mobile Arbeit“ ist weiter gefasst als der Rechtsbegriff „Telearbeit“ (Lott et al. 2021). Bei mobilem Arbeiten sind Beschäftigte an keinen Arbeitsplatz gebunden (Bundesministerium für Familien, Senioren, Frauen und Jugend 2017) und können Arbeit an mehreren oder wechselnden Einsatzorten, ortsungebundene Arbeit oder Arbeit an einem mobilen Arbeitsplatz verrichten (Beermann et al. 2018; Häring et al. 2018). Mobiles Arbeiten beschreibt eine Arbeit, die weder vom Büro noch vom Arbeitsplatz „zu Hause“ abhängig ist (Backhaus et al. 2021). Mobil gearbeitet werden kann auch nur an Teilen des Arbeitstages, wenn z. B. morgens im Büro und danach beim Kunden gearbeitet wird. Mobile Arbeit unterliegt grundsätzlich den Regelungen des Arbeitsschutzgesetzes und des Arbeitszeitgesetzes, spezielle Regelungen wie bei Telearbeit gibt es jedoch nicht (DGUV 2021).

Homeoffice kann als besondere Form des mobilen Arbeitens angesehen werden, als eine mögliche Option zur Arbeit am Arbeitsplatz im Büro. Beschäftigte führen ihre Tätigkeit, nach vorheriger Abstimmung mit der Organisation, zeitweilig im privaten Umfeld aus (DGUV 2021).

Telecommuting dagegen stellt einen Überbegriff dar, der sowohl Arbeit von Zuhause als auch von alternativen Orten einschließt (Gajendran und Harrison 2007).

Unter dem Begriff „orts- und zeitflexibles Arbeiten“ wird die Verlagerung der Tätigkeit an einen anderen Ort und die damit einhergehende zeitliche Flexibilisierung der Tätigkeit verstanden (Ahlers et al. 2021). Im Folgenden wird der Begriff „Homeoffice“ genutzt, um alle Formen der Büro-Arbeit von zu Hause während der Corona-Pandemie abzudecken.

In einer Studie von 2014 (Brenke 2016), die Daten aus Haushalts- und Personenumfragen wie den Mikrozensus oder das Sozio-oekonomische Panel beinhalteten, arbeiteten nur 12 % aller abhängig Beschäftigten überwiegend oder gelegentlich im Homeoffice, obgleich, basierend auf den Angaben der Befragten in der Studie, dies bei 40 % der Befragten bzw. deren Arbeitsplätze möglich wäre. Die Möglichkeiten zur Nutzung von Homeoffice variieren zwischen den Wirtschaftsbereichen: Überdurchschnittlich oft ist Homeoffice bei Tätigkeiten im Dienstleistungssektor möglich (insbesondere bei den Finanzdienstleistungen wie Banken), den Dienstleistungen für Unternehmen sowie in der öffentlichen Verwaltung – viel weniger häufig im Handel, im Verkehrsgewerbe sowie in Bereichen wie Gastgewerbe, Gesundheitswesen oder im Baugewerbe bzw. Landwirtschaft. Hier gibt es von der Art der Tätigkeiten her wenige Arbeitsplätze, die für Heimarbeit geeignet sind (Brenke 2016). Auch eine Arbeitszeitbefragung (Wöhrmann et al. 2020; Backhaus et al. 2020a) zeigt, dass 2019 nur 14 % der abhängig Beschäftigten im öffentlichen Dienst Telearbeit vereinbart hatten, während es 2015 nur 8 % und 2017 10 % waren. Die Zunahme an Beschäftigten mit der Möglichkeit zur Telearbeit zeigt sich auch in anderen Befragungen (z. B. Grunau et al. 2019). Mit der Corona-Pandemie hat Homeoffice auch im öffentlichen Dienst stark an Bedeutung gewonnen (Ahlers et al. 2021).

Viele Studien zum Thema Homeoffice wurden mit Personen durchgeführt, die schon Homeoffice-Erfahrungen sammeln konnten und teilweise seit Jahren im Homeoffice arbeiten (u. a. Bérastégui 2021). Auch Ahlers et al. (2021) zeigten, dass Homeoffice gerade dann als positiv erlebt wird, wenn es im Betrieb bereits Erfahrungen mit Homeoffice gibt und Regulierungen zur Ausstattung mit mobilen Geräten bzw. Zugriff auf interne Netze und Datenbanken vorhanden waren.

**Figure 1:**
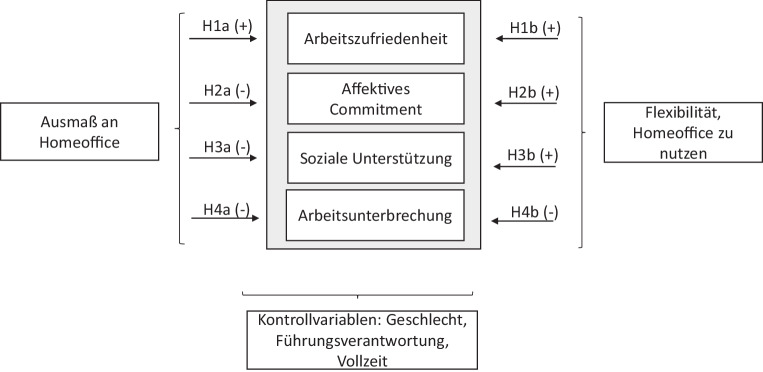

Es ist bisher nur spärlich untersucht worden, welche Folgen ein plötzlicher, z. B. pandemiebedingter Wechsel ins Homeoffice auf praxis- und arbeitsrelevante Variablen wie Arbeitszufriedenheit, Commitment, soziale Unterstützung und die Abgrenzung von äußeren Reizen (Arbeitsunterbrechungen) im öffentlichen Dienst hat. Welche Bedingungen der Arbeit im Homeoffice (z. B. Ausmaß an Homeoffice oder die Möglichkeit flexibel zu entscheiden, von wo man arbeiten möchte) sind entscheidend in Bezug auf arbeitsbezogene Variablen? Auch ist insbesondere im öffentlichen Dienst das Arbeiten im Homeoffice bisher wenig verbreitet (Brenke 2016). Vor diesem Hintergrund sollen die, aus der Praxis bedeutsamen, arbeitsrelevanten Variablen Arbeitszufriedenheit, Commitment, soziale Unterstützung und Arbeitsunterbrechungen in Zusammenhang mit dem Ausmaß an Homeoffice und mit der Möglichkeit, Homeoffice flexibel zu nutzen, genauer betrachtet werden. Die Untersuchung wird speziell für den öffentlichen Dienst durchgeführt, da hier nicht viele Studien und Erkenntnisse vorliegen (Neumann 2020). Die praxisrelevanten Konstrukte haben sich als wichtige Variablen in der Betrachtung von Homeoffice und vom Arbeiten im öffentlichen Dienst gezeigt (vgl. u. a. Backhaus et al. 2020a; Bonin et al. 2020; Gajendran und Harrison 2007). Abb. 1 zeigt einen Überblick der Zusammenhangshypothesen, die überprüft und im Folgenden erörtert werden sollen.

### Ausmaß der Arbeit im Homeoffice und Flexibilität zur Nutzung von Homeoffice

Viele Beschäftigte arbeiten nicht die volle Arbeitswoche im Homeoffice. Eine Arbeitszeitbefragung (Wöhrmann et al. 2020) zeigte, dass mehr als ein Drittel der Beschäftigten mit Telearbeitsvereinbarung weniger als einen Tag in der Woche telearbeiten, gefolgt von einem Tag pro Woche. Gajendran und Harrison (2007) stellten fest, dass die Intensität, also das Ausmaß an Homeoffice, eine Rolle spielt. Sie unterschieden high-intensity telecommuters (home-based), die ihre überwiegende Arbeitszeit außerhalb der zentralen Arbeitsstelle verbrachten und low-intensity telecommuters (office-based arrangement), welche die überwiegende Arbeitszeit am zentralen (konventionellen) Arbeitsort waren und Homeoffice nur an 1 bis 2 Tagen in der Woche nutzten. Auch Backhaus et al. (2020a) erscheint arbeitswissenschaftlich ein moderates Ausmaß an Telearbeit (z. B. ein Tag pro Woche) empfehlenswert zu sein.

Die Möglichkeit zum Homeoffice bringt für Beschäftigte u. a. eine erhöhte arbeitszeitbezogene Flexibilität und erhöhte (zeitliche) Autonomie sowie einen höheren Handlungsspielraum in Bezug auf die Planung und Einteilung der eigenen Arbeit mit sich (Gajendran und Harrison 2007; Grunau et al. 2019; Brownson 2004; Tavares 2017; Ulich und Wiese 2011; Brenke 2016; Wöhrmann et al. 2020). Zeitliche Flexibilitätsmöglichkeiten stellen Ressourcen für Beschäftigte dar, zum Beispiel um Arbeit und Privatleben besser zu vereinbaren (Backhaus et al. 2020b).

Homeoffice kann aber auch zu Vereinbarkeitsproblemen führen, wenn die Beschäftigten das Gefühl haben, nicht frei entscheiden zu können, ob sie im Homeoffice arbeiten (Kossek et al. 2006). Lapierre et al. (2016) zeigten in einer Studie mit Mitarbeitenden aus dem Finanzvertrieb, dass ein unfreiwilliges Arbeiten aus dem Homeoffice mit mehr stressbasierten Konflikten zwischen Arbeit und Privatleben einhergeht.

### Arbeitszufriedenheit

Ein wiederkehrendes Konstrukt in der Sozialforschung und ein relevantes Konstrukt in der Praxis am Büroarbeitsplatz ist die Zufriedenheit mit der eigenen Arbeit (u. a. Jochims 2019). Die bisherige Forschung zum Zusammenhang von der Arbeit im Homeoffice und der Arbeitszufriedenheit von Erwerbstätigen konnte zeigen, dass die Möglichkeit, von einem selbstbestimmten Ort zu arbeiten mit einer höheren Arbeitszufriedenheit einhergeht (z. B. Fonner und Roloff 2010; Masayuki 2018; Troup und Rose 2012; Wheatley 2017; Kaduk et al. 2019; Kossek et al. 2006). Grunau et al. (2019) stellten fest, dass Beschäftigte, die zumindest gelegentlich während der Arbeitszeit von zu Hause arbeiten, im Schnitt zufriedener sind als alle anderen Beschäftigten. Die mit Abstand am wenigsten zufriedenen Personen sind diejenigen, die gerne Homeoffice machen würden, dies aber nicht können. Auch Brenke (2016) konnte zeigen, dass Mitarbeitende, die sich Homeoffice wünschen, aber nicht die Möglichkeit dazu erhalten, unzufriedener mit ihrer Arbeit sind. Gajendran und Harrison (2007) ermittelten in ihrer Metaanalyse einen zwar geringen, aber günstigen Zusammenhang zwischen Homeoffice und Arbeitszufriedenheit, der vollständig von Autonomie mediiert wurde. Dies legt nahe, dass vor allem die Flexibilität zu entscheiden, von wo man arbeiten möchte, von Bedeutung ist.

Studien konnten zeigen, dass Homeoffice dazu beitragen kann, individuelle Bedürfnisse nach flexibler Arbeitsgestaltung zu realisieren und damit die Arbeitszufriedenheit der Beschäftigten zu verbessern (z. B. Bonin et al. 2020; Gajendran und Harrison 2007). Homeoffice geht mit Chancen und Risiken für die Gesundheit von Beschäftigten einher (Rothe et al. 2017): Eine Chance kann in der erhöhten zeitlichen und räumlichen Flexibilität liegen, welche die Vereinbarkeit von Familie und Beruf fördern kann.

Wir gehen daher davon aus, dass sowohl das Ausmaß an Homeoffice als auch der Grad an Flexibilität, Homeoffice zu nutzen einen voneinander unabhängigen Beitrag zur Aufklärung der Varianz der Arbeitszufriedenheit unter Einbezug von Kontrollvariablen leisten.

#### H1a

Ein höheres Ausmaß an Homeoffice geht mit einer höheren Zufriedenheit mit der Arbeit einher.

#### H1b

Eine größere Flexibilität zu entscheiden, ob man im Homeoffice arbeiten möchte, geht mit einer höheren Arbeitszufriedenheit einher.

### Affektives Commitment

Organisationales Commitment stellt ein weiteres zentrales Konzept der Arbeitsforschung dar und bezeichnet die Bindung eines Arbeitnehmers an sein Unternehmen, wobei das affektive Commitment die gefühlsmäßige Bindung bezeichnet (Allen und Meyer 1990). Affektives Commitment ist unter anderem positiv mit Organizational Citizenship Behavior (Ng und Feldman 2011) und negativ mit einem Austritt aus dem Arbeitsverhältnis assoziiert (Meyer et al. 2002).

Es ist wahrscheinlich, dass es einen gegenläufigen Zusammenhang zwischen dem Ausmaß an Homeoffice und affektivem Commitment einerseits, der Flexibilität und affektivem Commitment andererseits gibt. Je mehr Zeit Beschäftigte im Homeoffice verbringen, desto weniger Zeit verbringen sie mit Kolleginnen und Kollegen und Vorgesetzten, was die emotionale Bindung schwächen könnte. Andererseits gibt es Befunde, die nahelegen, dass ein höheres Ausmaß an Autonomie mit mehr affektivem Commitment einhergeht. Ramaswami et al. (1993) fanden positive Zusammenhänge zwischen Autonomie und affektivem Commitment. Es ist denkbar, dass eine Organisation ihre Mitarbeiter befähigt, indem sie ihnen die Autonomie gewährt, zu Hause zu arbeiten, und diese Befähigung das organisationale Engagement erhöht (z. B. Franz 2004; Peachey 2003).

Wir gehen daher von folgenden Hypothesen unter Einbezug von Kontrollvariablen aus:

#### H2a

Ein höheres Ausmaß an Homeoffice geht mit geringerem affektivem Commitment einher.

#### H2b

Eine größere Flexibilität zu entscheiden, ob man im Homeoffice arbeiten möchte, geht mit höherem affektivem Commitment einher.

### Soziale Unterstützung

Eine wichtige Variable in verschiedenen Modellen der Arbeitsforschung (u. a. Job Demand Control Support Model, Karasek und Theorell 1990) ist die soziale Unterstützung. Die Einbindung am Arbeitsplatz und die Unterstützung, die man durch die Arbeitskolleginnen und -kollegen und Führungskräfte erfährt, stellen wichtige Ressourcen für das Wohlbefinden von Beschäftigten dar (Abendroth und Reimann 2018; Vander Elst et al. 2017). Aber dennoch wird laut Grunau et al. (2019) dieses Konstrukt sowohl von Betrieben als auch von Beschäftigten häufig als Argument gegen Homeoffice genannt. Bei Anwesenheit am Arbeitsplatz können Beschäftigte einfacher miteinander kommunizieren und zusammenarbeiten. Daher stellt sich die Frage, ob das Arbeiten zu Hause zu einer Verschlechterung der Unterstützung zwischen Beschäftigten führt, aus „den Augen, aus dem Sinn“ (McCloskey und Igbaria 2003). Unter den Nutzerinnen und Nutzern von Homeoffice berichtet etwa jede/r Fünfte, dass der Kontakt zu Kolleginnen und Kollegen hierunter leidet (Grunau et al. 2019). In einer Studie bei Verwaltungen in Pandemiezeiten berichteten Beschäftigte im Homeoffice vor allem von Schwierigkeiten mit der Technik und von Kommunikationsdefiziten im Austausch mit den Arbeitskolleginnen und -kollegen (Next:Public 2020).

Ein hohes Ausmaß an Homeoffice kann dazu führen, dass direkter persönlicher Kontakt mit den Arbeitskolleginnen und -kollegen und Vorgesetzten weniger stattfindet bzw. der Austausch ausschließlich über technische Medien (wie Telefon, E‑Mail, Videokonferenzen) und auf fachliche Themen fokussiert bleibt (u. a. Golden und Gajendran 2019). Wöhrmann et al. (2020) fanden heraus, dass das Gefühl, bei der Arbeit Teil einer Gemeinschaft zu sein, bei Beschäftigten im Homeoffice weniger ausgeprägt ist und mit zunehmender Anzahl an Homeofficetagen abnimmt. Die Unterstützung durch Arbeitskolleginnen und -kollegen und Vorgesetzte scheint bei den Beschäftigten mit einem Tag pro Woche im Homeoffice am höchsten zu sein, sogar höher als bei ständiger Anwesenheit im Büro (u. a. Backhaus et al. 2020a). Gajendran und Harrison (2007) stellten in ihrer Metaanalyse fest, dass entgegen ihrer Erwartung Arbeit im Homeoffice positive Zusammenhänge mit der Beziehungsqualität zwischen Mitarbeitenden und Vorgesetzten aufwies, es für Personen mit einer hohen Intensität an Homeoffice jedoch negative Zusammenhänge bei der Beziehungsqualität mit den Kollegen und Kolleginnen gab.

Auch könnte der pandemiebedingt abrupt gestiegene Homeoffice-Anteil der Arbeitszeit und die fehlende Gewöhnung an diese Situation dazu führen, dass keine Möglichkeiten bestanden, bestimmte Regeln, Richtlinien oder andere Vorgehensweisen zu verabschieden, um die soziale Unterstützung uneingeschränkt zu gewährleisten. Deshalb gehen wir aufgrund der kurzfristigen Einführung von Homeoffice in der betrachteten Behörde vor dem Hintergrund der Pandemie von folgenden Hypothesen aus, unter Einbezug von Kontrollvariablen wie Führung:

#### H3a

Ein höherer Anteil an Homeoffice ist mit einer geringen wahrgenommenen sozialen Unterstützung verbunden.

Wir gehen davon aus, dass Beschäftigte, die flexibel entscheiden können, wann sie im Homeoffice arbeiten wollen, schon vor der Pandemie in der Organisation vermehrt Erfahrungen im Homeoffice gesammelt haben und dadurch auf informelle Netzwerke innerhalb der Organisation zurückgreifen können, die auch in Pandemiezeiten für soziale Unterstützung sorgen. Unterstützung für diese Annahme kommt von Ernst (2020), der in seiner Studie mit über 900 Teilnehmenden feststellte, dass Personen, die bislang noch nie im Homeoffice gearbeitet hatten, größere Probleme mit sozialer Isolation hatten als solche, die schon Homeofficeerfahrung aufwiesen. Daher nehmen wir an, dass ein mehr an Flexibilität mit höherer wahrgenommener sozialer Unterstützung einhergeht.

#### H3b

Eine größere Flexibilität zu entscheiden, von wo man arbeiten möchte, geht mit mehr wahrgenommener sozialer Unterstützung einher.

### Arbeitsunterbrechungen

Als eine zunehmende Arbeitsanforderung werden Arbeitsunterbrechungen gesehen (Baethge und Rigotti 2013). Viele Beschäftigte haben Arbeitsplätze, an denen ungestörtes Arbeiten oft nicht möglich ist; hier kann Homeoffice Störungen und Unterbrechungen reduzieren (vgl. van der Meulen et al. 2012). Wöhrmann et al. (2020) konnten zeigen, dass Beschäftigte mit zunehmender Anzahl an Homeofficetagen seltener von Störungen und Unterbrechungen bei der Arbeit betroffen sind. Personen, die im Homeoffice arbeiten, sind aufgrund weniger Arbeitsunterbrechungen zufriedener mit ihrer Berufstätigkeit (Fonner und Roloff 2010; Kröll et al. 2017; Kröll und Nüesch 2019). Homeoffice wird als passend für das konzentrierte Arbeiten eingeschätzt. Wenn Bürokonzepte wie z. B. activity based working, nicht optimal umgesetzt wurden, weil z. B. Rückzugsorte fehlen, so kann es sein, dass Mitarbeiter für konzentriertes Arbeiten in das Homeoffice ausweichen (Becker et al. 2021). Eine Voraussetzung ist hier jedoch, dass die Beschäftigten die Möglichkeit haben, selbst entscheiden zu können, ob sie in einer gegebenen Situation ins Homeoffice gehen möchten.

Es scheinen also sowohl das Ausmaß an Homeoffice als auch die Flexibilität und damit Entscheidungsmöglichkeit zum Rückzug, positive Effekte auf die Arbeitsunterbrechung zu haben. Deshalb gehen wir von folgenden Hypothesen unter Einbezug von Kontrollvariablen aus:

#### H4a

Ein höheres Ausmaß an Homeoffice hängt mit weniger Arbeitsunterbrechungen zusammen.

#### H4b

Eine größere Flexibilität zu entscheiden, ob man vom Homeoffice arbeiten möchte, geht mit weniger Arbeitsunterbrechungen einher.

## Methode

### Vorgehen und Stichprobe

Die Daten wurden über eine Onlinebefragung in einer Landesbehörde mit 1780 Mitarbeitenden (Stand zum Zeitpunkt der Befragung) in Baden-Württemberg durchgeführt. Die Erhebung erstreckte sich über drei Wochen im Dezember 2020 zur Zeit des „Lockdown light“. In der Behörde gab es bis zur Pandemie einen Anteil der Telearbeitenden von ca. 12 %. Die Beschäftigten wurden über das Intranet und direkte Ansprache von Vorgesetzten über die Befragung informiert. Der Fragebogen enthielt Fragen zu Homeoffice und Flexibilität, zu arbeitsbezogenen Aspekten, zur psychischen Gesundheit und zur Demografie. Da nur eine Behörde betrachtet wird, ist es möglich, eine homogene Stichprobe zu analysieren.

Der Link zur Befragung wurde 825 Mal angeklickt, und *n* = 484 Beschäftigte beendeten die Befragung, was einer Beendigungsquote von 58,7 % entspricht. Es wurden sieben Fälle entfernt, da sie bei für die Analysen relevanten demografischen Angaben fehlende Werte hatten. Die endgültige Stichprobe betrug somit *n* = 477 Personen. Davon waren *n* = 275 (57,7 %) weiblich; *n* = 60 Teilnehmende waren bis 30, *n* = 110 zwischen 31 und 40, *n* = 98 zwischen 41 und 50, *n* = 156 zwischen 51 und 60 und *n* = 53 über 60 Jahre alt. Von den Teilnehmenden hatten *n* = 121 (24,4 %) Führungsverantwortung, und *n* = 342 (71,7 %) arbeiteten in Vollzeit.

In der Befragung wurde die gesamte Bandbreite von keiner bis täglicher Homeoffice-Nutzung (= Ausmaß an Homeoffice) erfasst. Während *n* *=* *107* (22,1 %) nach eigener Angabe nie im Homeoffice arbeitet, arbeiteten die restlichen Befragten durchschnittlich 35,8 % ihrer Arbeitszeit von zuhause. Unter den Befragten können *n* *=* *384 *(79,5 %) zumindest gelegentlich selbst entscheiden, wo sie arbeiten möchten (flexible Nutzung von Homeoffice). Von den Teilnehmenden hatten *n* *=* *99* (20,5 %) diese Wahlmöglichkeit kaum bzw. nie.

### Erhebungsinstrumente

Sofern nicht anders angegeben, wurden die Variablen mit einer fünfstufigen Likertskala mit den Polen 1 (*trifft überhaupt nicht zu*) bis 5 (*trifft völlig zu*) erfasst.

#### Ausmaß an Homeoffice

Das Ausmaß an Homeoffice wurde auf Basis der durchschnittlichen wöchentlichen Arbeitsstunden im Homeoffice und der Gesamtarbeitszeit errechnet, indem wir den Anteil der Stunden im Homeoffice an der Gesamtarbeitszeit berechneten. Hier wurde nach den Erfahrungen der letzten 3 Monate gefragt.

#### Flexibilität zur Nutzung von Homeoffice

Der Grad an Flexibilität wurde mit dem Item „Können Sie selbst entscheiden, an welchem Ort (Büro, zu Hause etc.) Sie arbeiten?“ erfragt (Bauer et al. 2014). Die Antwortskala war fünfstufig und reichte von 1 (*nie/sehr selten*) bis 5 (*sehr oft/immer*).

#### Arbeitszufriedenheit

Die Arbeitszufriedenheit wurde mit drei Items von Cammann et al. (1983) in der deutschen Übersetzung von Ivens (2018) erfasst. In der Stichprobe von Ivens (2018) betrug Cronbachs Alpha α = 0,87, in unserer Stichprobe α = 0,77.

#### Affektives Commitment

Affektives Commitment wurde mit der Skala von Allen und Meyer (1990) in der deutschen Übersetzung von Schmidt et al. (1998) erhoben. Die acht Items wurden auf einer siebenstufigen Likertskala mit den Polen 1 (*stimme überhaupt nicht zu*) bis 7 (*stimme vollständig zu*) beantwortet. Cronbachs Alpha betrug in den Validierungsstudien von Allen und Meyer (1990) α = 0,86 bzw. α = 0,87. In unserer Stichprobe betrug α = 0,81.

#### Soziale Unterstützung

Soziale Unterstützung wurde mit der Community-Subskala der Areas of Worklife Scale (Leiter und Maslach 1999) in der deutschen Übersetzung von Schulze et al. (2013) erhoben. In einer Validierungsstudie (Brom et al. 2015) betrug Cronbachs Alpha zwischen α = 0,81 und α = 0,84. In unserer Stichprobe betrug α = 0,85.

#### Arbeitsunterbrechungen

Arbeitsunterbrechungen wurden mit drei adaptierten Items aus der Subskala Arbeitsunterbrechung des Instrument zur stressbezogenen Tätigkeitsanalyse ISTA (Semmer et al. 1999) erhoben. Cronbachs Alpha betrug α = 0,82.

#### Kontrollvariablen

Als Kontrollvariablen haben wir Geschlecht (u. a. Schieman und Glavin 2008), Arbeitsumfang (Voll- bzw. Teilzeit, weniger als 39 Wochenstunden) und Führungsverantwortung der Befragten aufgenommen. Vor allem Führungsverantwortung ist bedeutsam, da Führungskräfte sowohl einen höheren Grad an Flexibilität haben, wo sie arbeiten wollen und in der Regel gleichzeitig höhere Werte bei arbeitsbezogenen Einstellungskonstrukten aufweisen (u. a. Bundesministerium für Familien, Senioren, Frauen und Jugend 2017; Bonin et al. 2020; Hattendorf et al. 2015). Jede dieser Variable wurde dichotom erfasst, jeweils mit einem Item.

### Statistische Analysen

Alle Berechnungen wurden mit SPSS 26 durchgeführt. Für jede der Hypothesen (jeweils a und b‑Teil zusammen) wurde eine multiple Regressionsanalyse durchgeführt. Dabei waren Arbeitszufriedenheit, Arbeitsunterbrechungen, soziale Unterstützung und affektives Commitment die Kriteriumsvariablen und bei jeder der Analysen das Ausmaß an Homeoffice, Grad an Flexibilität zur Nutzung von Homeoffice, Geschlecht, Vollzeit/Teilzeit und Führungsverantwortung die Prädiktoren, so dass bei jeder Analyse die mögliche Varianzüberschneidung von Homeoffice und Flexibilität sowie der Kontrollvariablen mit einbezogen wurde.

## Ergebnisse

Die Mittelwerte, Standardabweichungen und Interkorrelationen aller Variablen finden sich in Tab. 1.

**Table 1:** 

	*M*	1	2	3	4	5	6	7	8	9
Ausmaß Homeoffice	0,36	–	–	–	–	–	–	–	–	–
Flexibilität Homeoffice	3,48	0,35***	–	–	–	–	–	–	–	–
Arbeitszufriedenheit	4,36	−0,01	0,18***	**0,77**	–	–	–	–	–	–
Arbeitsunterbrechungen	3,13	−0,13**	0,02	0,01	**0,82**	–	–	–	–	–
Commitment	4,44	0,04	0,10*	0,51***	0,09	**0,81**	–	–	–	–
Soziale Unterstützung	3,84	−0,00	0,13**	0,46***	0,04	0,39***	**0,85**	–	–	–
Geschlecht	–	0,08	−0,19***	−0,01	−0,02	0,01	−0,06	–	–	–
Führungsverantwortung	–	0,06	−0,10*	−0,18***	−0,24***	−0,22***	−0,16***	0,12**	–	–
Vollzeit	–	0,08	0,02	0,04	−0,05	−0,01	−0,02	0,34***	0,15	–

Mittelwerte und Interkorrelationen im imputierten Datensatz; Cronbachs Alpha in der Diagonale; Geschlecht 1 = männlich, 2 = weiblich; Führungsverantwortung 1 = ja, 2 = nein; Vollzeit 1 = ja, 2 = nein

*p < 0,05, **p < 0,01, ***p < 0,001

Der durchschnittliche Anteil von Homeoffice an der Gesamtarbeitszeit betrug trotz der Pandemie nur etwas über ein Drittel; fast 80 % der Befragten nutzen jedoch zumindest manchmal Homeoffice. Die Einschätzung der Flexibilität lag über dem Skalenmittelwert, aber die Standardabweichung indiziert eine hohe Variabilität zwischen den Beschäftigten.

### Vorbereitende Analysen

Zunächst untersuchten wir die fehlenden Werte im Datensatz. Insgesamt fehlten rund 0,5 % der Werte und bei keiner Variablen mehr als 3 %. Da das Ergebnis des MCAR-Tests nach Little signifikant war, χ^2^ = 3110,501, *p* < 0,001, entschlossen wir uns dazu, die fehlenden Werte multipel zu imputieren. Wir imputierten auf Itemebene, und es entstanden fünf vollständige Datensätze, die sich in den Kennzahlen nur geringfügig unterschieden. Die Zahl der Fälle reduzierte sich von *n* = 284 auf *n* = 277, da wir keine fehlenden Werte bei demografischen Variablen ersetzten.

Linearität, Normalverteilung der Residuen, Multikolinearität und Homoskedastizität waren unproblematisch. Allerdings identifizierten wir für jede Analyse multivariate Ausreißer über die standardisierten Residuen, die Hebelwerte und die Cook’sche Distanz. Wir rechneten daher alle Analysen mit und ohne Ausreißer. Da sich in keinem Fall bedeutsame Abweichungen zwischen den Analysen mit und ohne Ausreißer ergaben, berichten wir nur die Ergebnisse mit Ausreißern.

### Hauptanalysen

Details zu den Regressionsanalysen finden sich in den Tab. 2, 3, 4 und 5. Berichtet werden hier stets die mittleren Werte aus den fünf imputierten Datensätzen.

**Table 2:** 

	*B*	*SE B*	β	*p*
Ausmaß Homeoffice	−0,16	0,10	−0,08	0,11
Flexibilität Homeoffice	0,09	0,02	0,19	0,00
Geschlecht	0,04	0,06	0,03	0,51
Führungsverantwortung	−0,24	0,06	−0,17	0,00
Vollzeit	0,08	0,06	0,06	0,22

Durchschnitt über alle Imputationen hinweg; *R*^2^ = 0,07, *p* < 0,001; Geschlecht 1 = männlich, 2 = weiblich; Führungsverantwortung 1 = ja, 2 = nein; Vollzeit 1 = ja, 2 = nein

**Table 3:** 

	*B*	*SE B*	β	*p*
Ausmaß Homeoffice	0,06	0,17	0,02	0,72
Flexibilität Homeoffice	0,06	0,04	0,07	0,14
Geschlecht	0,08	0,10	0,04	0,40
Führungsverantwortung	−0,50	0,11	−0,22	0,00
Vollzeit	0,02	0,11	0,01	0,89

Durchschnitt über alle Imputationen hinweg; *R*^2^ = 0,05, *p* < 0,001; Geschlecht 1 = männlich, 2 = weiblich; Führungsverantwortung 1 = ja, 2 = nein; Vollzeit 1 = ja, 2 = nein

**Table 4:** 

	*B*	*SE B*	β	*p*
Ausmaß Homeoffice	−0,05	0,13	−0,02	0,67
Flexibilität Homeoffice	0,06	0,03	0,11	0,02
Geschlecht	−0,03	0,08	−0,02	0,70
Führungsverantwortung	−0,24	0,08	−0,14	0,00
Vollzeit	−0,00	0,08	−0,00	0,98

Durchschnitt über alle Imputationen hinweg; *R*^2^ = 0,04, *p* < 0,01; Geschlecht 1 = männlich, 2 = weiblich; Führungsverantwortung 1 = ja, 2 = nein; Vollzeit 1 = ja, 2 = nein

**Table 5:** 

	B	SE B	β	*p*
Ausmaß Homeoffice	−0,41	0,15	−0,14	0,01
Flexibilität Homeoffice	0,03	0,03	0,05	0,31
Geschlecht	0,06	0,09	0,03	0,52
Führungsverantwortung	−0,46	0,09	0,22	0,00
Vollzeit	−0,04	0,09	−0,02	0,67

Durchschnitt über alle Imputationen hinweg; R^2^ = 0,07, *p* < 0,001; Geschlecht 1 = männlich, 2 = weiblich; Führungsverantwortung 1 = ja, 2 = nein; Vollzeit 1 = ja, 2 = nein

#### Arbeitszufriedenheit

In unseren Hypothesen gingen wir davon aus, dass sowohl ein höherer Anteil an Homeoffice (H1a) als auch ein höherer Grad an Flexibilität (H1b) unter Einbezug des jeweils anderen Konstruktes und Kontrollvariablen mit mehr Arbeitszufriedenheit einhergehe. Das Regressionsmodell war signifikant, R^2^ = 0,07, *p* < 0,001. Das Ausmaß von Homeoffice leistete wider Erwarten keinen statistisch signifikanten Beitrag zur Aufklärung der Varianz der Arbeitszufriedenheit, β = −0,08, *p* = 0,11, während der Grad an Flexibilität zur Nutzung von Homeoffice dies tat, β = 0,19, *p* < 0,001.

#### Affektives Commitment

Wir nahmen an, dass ein höherer Anteil an Homeoffice (H2a) mit weniger affektivem Commitment einhergehe, ein höherer Grad an Flexibilität (H2b) aber mit mehr affektiven Commitment jeweils unter Einbezug des anderen Konstruktes sowie Kontrollvariablen. Bivariat konnten wir den angenommenen Zusammenhang zwischen Flexibilität und affektivem Commitment finden, *r* = 0,10, *p* < 0,05. Allerdings bestätigte sich das Ergebnis nicht unter Einbezug der Kontrollvariablen. Das Regressionsmodel war zwar signifikant, R^2^ = 0,05, *p* < 0,001, entgegen unserer Annahmen leistete jedoch weder das Ausmaß an Homeoffice, β = 0,02, *p* = 0,72, noch der Grad an Flexibilität zur Nutzung von Homeoffice, β = 0,07, *p* = 0,14, einen signifikanten Beitrag zur Aufklärung der Varianz des affektiven Commitments, so dass die Hypothesen abgelehnt werden. Führungsverantwortung stellte sich als signifikanter Prädiktor von affektiven Commitment heraus, β = −0,22, *p* < 0,001.

#### Soziale Unterstützung

Wir vermuteten, dass unter Einbezug des jeweils anderen Konstruktes und von Kontrollvariablen ein höheres Ausmaß an Homeoffice mit weniger wahrgenommener sozialer Unterstützung einhergehe (H3a), während wir einen positiven Zusammenhang zwischen Grad an Flexibilität zur Nutzung von Homeoffice und wahrgenommener sozialer Unterstützung annahmen (H3b). Das Regressionsmodell wurde signifikant, R^2^ = 0,04, *p* < 0,01. Anders als vorhergesagt, war das Ausmaß an Homeoffice jedoch kein signifikanter Prädiktor, β = −0,04, *p* = 0,43, während sich die Hypothese zur Flexibilität bestätigte, β = 0,13, *p* < 0,05.

#### Arbeitsunterbrechungen

Wir nahmen an, dass sowohl das Ausmaß an Homeoffice (H4a) als auch der Grad an Flexibilität zur Nutzung von Homeoffice (H4b) mit weniger Arbeitsunterbrechungen einhergehe. Das Regressionsmodell war signifikant, R^2^ = 0,07, *p* < 0,001 und wie vorhergesagt, ging ein höheres Ausmaß an Homeoffice mit weniger Arbeitsunterbrechungen einher, β = −0,14, *p* < 0,01. Der Grad der Flexibilität war nicht signifikant, β = 0,05, *p* = 0,31.

## Diskussion

Die Arbeit im Homeoffice in deutschen Betrieben hat während der Corona-Pandemie an Bedeutung gewonnen. Etwa ein Fünftel der Betriebe, in welchen die Tätigkeiten grundsätzlich eine Arbeit von zuhause zulassen, plant aufgrund der Erfahrungen während der Corona-Krise, Möglichkeiten zum Homeoffice bzw. zur Telearbeit zukünftig auszubauen (Backhaus et al. 2020a). Auch für den öffentlichen Dienst spielt Homeoffice inzwischen eine Rolle. In diesem Artikel wollten wir für Beschäftigte einer Behörde feststellen, ob Arbeitsvariablen (Arbeitszufriedenheit, affektives Commitment, soziale Unterstützung und Arbeitsunterbrechungen) in Zusammenhang stehen mit dem Ausmaß an Homeoffice und mit der Flexibilität zur Nutzung von Homeoffice.

Drei der acht Hypothesen bestätigten sich. In Einklang mit bisherigen Forschungsergebnissen (u. a. Gajendran und Harrison 2007) ging der Grad an Flexibilität, Homeoffice nach Bedarf zu nutzen, mit mehr Arbeitszufriedenheit einher. Ein höheres Ausmaß an Homeoffice leistete keinen bedeutsamen Beitrag zur Erklärung der Arbeitszufriedenheit. Nur allein die Nutzung von Homeoffice, egal ob ein größeres oder geringeres Ausmaß, scheint nicht zu einer höheren Arbeitszufriedenheit beizutragen, sondern die Möglichkeit, selbst flexibel entscheiden zu können von wo gearbeitet wird, was eine Form der Autonomie darstellt und mit den klassischen Modellen zur Erklärung von Arbeitszufriedenheit vereinbar ist (z. B. Job-Characteristics-Modell, Hackman und Oldham 1976; Job Demands-Resources-Model, Bakker und Demerouti 2007).

Entgegen unserer Annahmen spielen weder das Ausmaß an Homeoffice noch der Grad an Flexibilität zur Nutzung von Homeoffice eine Rolle beim affektiven Commitment. Im Übrigen fand sich die Führungsverantwortung als bedeutsamer Prädiktor von affektiven Commitment. Wir fanden zwar einen statistisch bedeutsamen bivariaten Zusammenhang in der erwarteten Richtung zwischen Grad an Flexibilität und affektiven Commitment. Die Tatsache, dass dieser Zusammenhang nach Hinzunahme der Kontrollvariablen nicht mehr signifikant war, könnte dadurch zustande kommen, dass Führungskräfte sowohl ein höheres Maß an Flexibilität sowie ein höheres Ausmaß an affektiven Commitment aufweisen, dadurch ein Teil der gemeinsamen Varianz zwischen Flexibilität und affektiven Commitment durch die Rolle als Führungskraft erklärt wird und diese insgesamt bedeutsamer als Homeoffice für das affektive Commitment ist.

Anders als vorhergesagt, scheint ein höheres Ausmaß an Homeoffice keinen negativen Einfluss auf die wahrgenommene soziale Unterstützung zu haben, während der Grad an Flexibilität zur Nutzung von Homeoffice positiv mit sozialer Unterstützung zusammenhängt. Die Ergebnisse sprechen dafür, dass Homeoffice nicht die Pflege der Kontakte und damit die Zusammenarbeit behindert. Oft wird bei der Arbeit von zu Hause von einer Verschlechterung der Zusammenarbeit zwischen Beschäftigten ausgegangen, obwohl unter den Nutzenden von Homeoffice etwa jede/r Fünfte berichtet, dass der Kontakt zu Kolleginnen und Kollegen hierunter leidet (Grunau et al. 2019). Die Anwesenheit am Arbeitsplatz bringt zwar den Vorteil mit sich, dass Beschäftigte einfacher miteinander kommunizieren und zusammenarbeiten können; unsere Ergebnisse zeigen aber, dass dies nicht von der Anzahl an Homeofficetagen abhängt. Der positive Zusammenhang von sozialer Unterstützung mit dem Ausmaß an Flexibilität könnte darauf hindeuten, dass die wahrgenommene soziale Unterstützung durch die Flexibilität gestärkt wird, denn wenn einem Arbeitgeber Flexibilität eingeräumt wird, ist dies im weiteren Sinne bereits eine Form von sozialer Unterstützung durch den Vorgesetzten bzw. durch die Organisation; der positive Zusammenhang von sozialer Unterstützung mit dem Ausmaß an Flexibilität könnte andererseits aber auch auf einen Halo-Effekt hindeuten.

Erwartungskonform ging ein höheres Ausmaß an Homeoffice mit weniger Arbeitsunterbrechungen einher. Dieses Ergebnis ist mit der Literatur konform, in der sich zeigte, dass die Arbeit im Homeoffice die Möglichkeit bietet, konzentriert und ohne Unterbrechung zu arbeiten. Beschäftigte mit zunehmender Anzahl an Homeofficetagen sind seltener von Störungen und Unterbrechungen bei der Arbeit betroffen (Wöhmann et al. 2020). Der Grad an Flexibilität zur Nutzung von Homeoffice spielte für Arbeitsunterbrechungen keine Rolle.

Zusammenfassend konnten die Ergebnisse der Untersuchung zeigen, dass Homeoffice auch während der Pandemie keine nachteiligen Auswirkungen auf die untersuchten Konstrukte hat. Wenn das Konstrukt Arbeitsunterbrechung betrachtet wird, ist die „Quantität“ (Ausmaß an Homeoffice) relevant. Jedoch zeigt sich für die anderen drei arbeitsbezogenen Konstrukte Arbeitszufriedenheit, affektives Commitment und soziale Unterstützung, dass nicht die „Quantität“ des Homeoffices bedeutsam ist, sondern die „Qualität“, in diesem Fall die Wahlmöglichkeit zur Nutzung von Homeoffice. Die vorliegenden Befunde liefern Evidenz für die Bedeutsamkeit der flexiblen Nutzung von Homeoffice und damit der wahrgenommenen Möglichkeit, selbst darüber zu entscheiden, ob im Homeoffice gearbeitet wird.

### Methodische Einschränkungen

Die Studie weist sowohl Stärken als auch Schwächen auf. Dadurch, dass sowohl der Grad an Flexibilität wie auch das tatsächliche Ausmaß an Homeoffice und Kontrollvariablen berücksichtigt wurden, konnte ein differenzierteres Bild davon gezeichnet werden, inwiefern die tatsächliche Arbeit im Homeoffice und/oder die Möglichkeit hierzu mit erstrebenswerten Folgen zusammenhängt. Ebenfalls als Stärken sind die große und lebensechte Stichprobe (d. h. keine Studierenden, keine als-ob Untersuchung) zu nennen. Andererseits gibt es auch methodische Einschränkungen: Es handelt sich um eine querschnittliche Untersuchung, die keine gesicherte kausale Interpretation der gefundenen Zusammenhänge erlaubt. Zukünftige Forschung sollte längsschnittliche Untersuchungen anstreben, um eine ursächliche Wirkung zu überprüfen und gegebenenfalls auch Gewöhnungseffekte festzustellen. Die beiden Maße „Ausmaß an Homeoffice“ und „Grad der Flexibilität der Nutzung von Homeoffice“ fußen auf jeweils einem Item, was hinsichtlich psychometrischer Anforderungen suboptimal war. Jedoch war es in der Befragung nicht möglich, die Bearbeitungszeit zu verlängern, was eine so knappe Operationalisierung der Konstrukte erforderte. Ohly et al. (2010) schlagen vor, jeweils ein Item pro Konstrukt auszuwählen, welches dieses am besten repräsentiert. Weiterhin basieren alle Variablen dieser Untersuchung auf der Selbsteinschätzung der Beschäftigten, so dass geteilte Varianz zwischen den Konstrukten auf das Vorliegen ein- und derselben Urteilsquelle zurückgeführt werden könnte (Podsakoff et al. 2003). Darüber hinaus waren die Teilnehmenden aus einer Behörde; dies könnte die Übertragbarkeit auf andere Behörden einschränken. Die Generalisierbarkeit für andere Bereiche müsste also in weiteren Untersuchungen geprüft werden. Schließlich fand die Erhebung unter dem Einfluss der Corona-Pandemie statt, was aber nicht nur eine Beschränkung darstellt, sondern im Sinne eines natürlichen Experiments erstmals die Untersuchung der Zusammenhänge von unversehens stattfindender Arbeit im Homeoffice und verschiedenen Arbeitskonstrukten erlaubte. Die möglicherweise von anderen Aspekten der Corona-Pandemie gefärbten Ergebnisse sollten zukünftig unter Normalbedingungen noch einmal überprüft werden.

### Implikationen und Ausblick

Zusammenfassend kann man für die zukünftige Ausgestaltung von Homeoffice festhalten, dass Homeoffice keine nachteiligen Auswirkungen auf die soziale Unterstützung zu haben scheint. Das bloße Ausmaß an Homeoffice ist allerdings nicht entscheidend dafür, ob Mitarbeitende zufriedener mit der Arbeit sind, obwohl im Homeoffice ungestörteres Arbeiten oft möglich ist. Ein wichtiges Gestaltungsmerkmal von Homeoffice ist die Flexibilität des Arbeitsortes, d. h. selbst entscheiden zu können, von welchem Ort aus man arbeitet. Tatsächlich zeigt die vorliegende Untersuchung, dass Flexibilität bei der Wahl des Arbeitsortes mit arbeitsbezogenen Variablen in positivem Zusammenhang steht. Personen, die in höherem Grade über ihren Arbeitsort selbst bestimmen können, sind zufriedener mit der Arbeit und berichten eine höhere soziale Unterstützung.

All dies impliziert, dass Organisationen – Behörden inbegriffen – den Beschäftigten eine möglichst große Flexibilität und damit Handlungsspielraum bei der Ausgestaltung flexibler Arbeitsmöglichkeiten geben und starre Regeln vermeiden sollten. Es muss nicht unbedingt ein hoher Umfang von Homeoffice angeboten werden, sondern es sollte die Ausgestaltung von Homeoffice zugunsten der Flexibilität, z. B. von Präsenz- und Homeoffice-Tagen, erfolgen. Diese Flexibilität, also Wahlmöglichkeit, ist eine Ressource für die Mitarbeitenden. Wenn die Mitarbeitenden selbst entscheiden können, wie sie die Möglichkeit von Homeoffice einsetzen und damit die Vorzüge des Homeoffices für sich nutzbar machen, könnte dies auch der Bewältigung von Arbeitsanforderungen dienen, etwa im Sinne einer Situationskontrolle im secondary coping des transaktionalen Stressmodells von Lazarus und Folkman (Biggs et al. 2017). Damit können die Beschäftigten einen positiven Einfluss auf ihre Arbeitssituation ausüben.

Über unsere Studie hinausgehend sieht es jedoch so aus, dass Homeoffice nur einen Teil der Arbeit ausmacht und eine der (Arbeitsplatz‑)Optionen bleiben sollte. Studien konnten zeigen, dass die Arbeitszufriedenheit bei Beschäftigten höher ist, die nur gelegentlich und nicht regelmäßig von zu Hause oder mobil arbeiten (u. a. Golden und Veiga 2005; Stettes 2016). Das betriebliche Büro bleibt weiterhin wichtig als „Hub und Home“: Wenn Anforderungen und Prozesse komplexer und dynamischer werden, sind vernetzter Austausch, flexible Arbeitsweisen und stabile Arbeitsbeziehungen entscheidend, und all dies wird schwierig ohne unmittelbaren Austausch im betrieblichen Büro (Kratzer 2020). Auch Alipour et al. (2020) gehen davon aus, dass der durch die Pandemie ausgelöste Homeoffice-Trend nachhaltige Auswirkungen auf die Organisation von Arbeit haben könnte, aber es dennoch sein kann, dass Betriebe und Beschäftigte eine hybride Arbeitsform zwischen Homeoffice und Präsenzarbeit anstreben, so dass Büroarbeitsplätze Treffpunkte für die Mitarbeitenden werden.

Eine ausgeprägte Homeofficekultur in Organisationen ist gekennzeichnet durch Vertrauen in die Beschäftigten, eine wertschätzende Kommunikation sowie eine gute Organisation von Zielvereinbarungen und Regelungen bezüglich der Erreichbarkeit und der Arbeitsaufgaben im Homeoffice (Initiative Neue Qualität der Arbeit 2020). Um Arbeitsmotivation und Engagement von Beschäftigten zu wahren, ist ein regelmäßiger und effektiver Informationsaustausch, insbesondere auch mit den Führungskräften, wichtig (siehe Weinert et al. 2015; De Vries et al. 2018).

Mitarbeitende, die viel im Homeoffice arbeiten, können ungestörter, ohne Arbeitsunterbrechungen, arbeiten. Es liegt aber auch mehr Verantwortung bei den Beschäftigten im Homeoffice, u. a. die Kommunikation betreffend. Es macht daher Sinn, die Beschäftigten zu einer aktiven Kommunikation – gerade aus dem Homeoffice heraus – zu ermutigen. Untersuchungen zeigen verschiedene Wege auf, um die Zusammenarbeit und Kommunikation sicherzustellen, z. B. durch die Bereitstellung von Sofortnachrichten-Programmen oder anderen Kommunikationstechnologien (u. a. ver.di 2019) und auch Telefon- und Videokonferenzen (Klaffke 2016).

Nicht nur im Homeoffice sollte ein ungestörtes Arbeiten ermöglicht werden. Die Nutzung von (Arbeitsplatz‑)Optionen wie unterschiedliche Rückzugsräume aber auch Spielregeln bzw. Verhaltensempfehlungen, die den Umgang mit Störungen betreffen, können gegen Arbeitsunterbrechung helfen. Je mehr Optionen der Raumnutzung, mit der Möglichkeit, auch im Homeoffice zu arbeiten, Beschäftigte haben, desto zufriedener sind sie; und je klarer die Spielregeln sind, desto besser das Zusammenleben im Raum (Kratzer 2020). Eine gemeinsam genutzte Arbeitsfläche funktioniert nicht ohne Regeln, die gemeinsam erarbeitet, kommuniziert und nachgehalten werden müssen (Becker et al. 2021). Und Spielregeln bzw. Verhaltensempfehlungen können auch allgemein auf den Umgang mit Homeoffice und die Gleichbehandlung unterstützend wirken. In der neuen Arbeitswelt ist es wichtig, sowohl die individuelle als auch die kollektive Kompetenz zu nutzen (Becker et al. 2021). Die Team- aber auch die Organisationsebene sollte daher nicht außer Acht gelassen werden, da die Team-Resilienz aber auch Organisation-Resilienz eine entscheidende Kompetenz darstellen kann, die Zusammenarbeit zu optimieren (u. a. Stoverink et al. 2020).

### Fazit

Die Erfahrungen mit Homeoffice waren für Mitarbeitende von Behörden eher positiv: Während vor der Pandemie Homeoffice nur begrenzt möglich war und im Positivfalle eher langwierige Prozesse implizierte, mussten die Behörden während der Pandemie schnell reagieren (Next:Public 2020). Der öffentliche Dienst scheint in einem Wandel begriffen, bei dem Arbeitsergebnisse und weniger die Präsenz von Beschäftigten im Vordergrund stehen (Neumann et al. 2020). Die Zukunft der Telearbeit ist an einem wichtigen Punkt angelangt, denn Arbeitgeber erkennen zunehmend die Vorteile solcher Vereinbarungen (Bérastégui 2021).

Die Möglichkeit, entscheiden zu können, von wo man arbeitet, stellt eine Ressource dar. Die Flexibilität des Arbeitsortes, d. h. selbst entscheiden zu können, von welchem Ort aus man arbeitet, steht im positiven Zusammenhang mit wichtigen arbeitsbezogenen Variablen. Eine solche Flexibilität kann ebenfalls den Ausschlag dafür geben, dass sich Bewerber für die Organisation entscheiden und allgemein dafür, dass sich die Attraktivität der Arbeitsstelle erhöht. Überhaupt kann der Ausbau der mobilen Arbeit dem Fachkräftemangel ein Stück weit entgegenwirken, denn ortsflexibles Arbeiten ermöglicht den Zugang zu Mitarbeitenden in größerer räumlicher Entfernung (Lott et al. 2021). Auch nach der Pandemie sollte Homeoffice mit Augenmerk daher zur Normalität werden.

## Footnotes

1 Activity-Based Offices zeichnen sich durch ein offenes und flexibles Raumkonzept aus: Ein Großteil der Arbeitsplätze befindet sich in offenen Bereichen ohne Zwischenwände. Es handelt sich um ein Bürokonzept mit unterschiedlichen Arbeitsorten für unterschiedliche Arbeitsaufgaben, für den Austausch aber auch Rückzugsmöglichkeiten wie Telefonzellen (Becker et al. 2021). Activity-based offices gelten als Antwort auf eine beschleunigte Innovationsdynamik, die wachsenden Flexibilitätserfordernisse sowie die Informationsflut einer digitalisierten Wissensgesellschaft (Kratzer 2020).
